# Intracellular Nucleic Acid Delivery by the Supercharged Dengue Virus Capsid Protein

**DOI:** 10.1371/journal.pone.0081450

**Published:** 2013-12-05

**Authors:** João Miguel Freire, Ana Salomé Veiga, Thaís M. Conceição, Wioleta Kowalczyk, Ronaldo Mohana-Borges, David Andreu, Nuno C. Santos, Andrea T. Da Poian, Miguel A. R. B. Castanho

**Affiliations:** 1 Instituto de Medicina Molecular, Faculdade de Medicina, Universidade de Lisboa, Lisbon, Portugal; 2 Instituto de Bioquímica Médica, Universidade Federal do Rio de Janeiro, Rio de Janeiro, Brazil; 3 Department of Experimental and Health Sciences, Pompeu Fabra University, Barcelona Biomedical Research Park, Barcelona, Spain; 4 Instituto de Biofísica Carlos Chagas Filho, Universidade Federal do Rio de Janeiro, Rio de Janeiro, Brazil; Institut National de la Santé et de la Recherche Médicale, France

## Abstract

Supercharged proteins are a recently identified class of proteins that have the ability to efficiently deliver functional macromolecules into mammalian cells. They were first developed as bioengineering products, but were later found in the human proteome. In this work, we show that this class of proteins with unusually high net positive charge is frequently found among viral structural proteins, more specifically among capsid proteins. In particular, the capsid proteins of viruses from the *Flaviviridae* family have all a very high net charge to molecular weight ratio (> +1.07/kDa), thus qualifying as supercharged proteins. This ubiquity raises the hypothesis that supercharged viral capsid proteins may have biological roles that arise from an intrinsic ability to penetrate cells. Dengue virus capsid protein was selected for a detailed experimental analysis. We showed that this protein is able to deliver functional nucleic acids into mammalian cells. The same result was obtained with two isolated domains of this protein, one of them being able to translocate lipid bilayers independently of endocytic routes. Nucleic acids such as siRNA and plasmids were delivered fully functional into cells. The results raise the possibility that the ability to penetrate cells is part of the native biological functions of some viral capsid proteins.

## Introduction

Nucleic acid- or protein-based therapies are limited by the difficult cellular internalization of these macromolecules [1], [2]. Several delivery systems have been developed and proposed to improve the viability of these molecular therapies. Examples of these systems can range from cell electroporation to viral delivery [3], [4], or the use of liposomes [5], [6], inorganic compounds (cationic polymers, nanotubes, nanoparticles) and cell-penetrating peptides (CPPs) as delivering systems [7]–[11]. Recently, studies from David Liu’s lab reported that supercharged proteins – SCPs – (or supercharging a regular protein by means of amino acid mutations) may serve as potent drug delivery systems into a wide variety of mammalian cells and tissues [12]–[15]. The first of this novel class of proteins was created by direct mutagenesis of solvent exposed-amino acid residues of the green fluorescent protein (GFP), which resulted in a GFP with a global net charge of +48 (*wt* is −30), with minimum aggregation propensity and potent cell-penetrating ability [12], [16]. The cell-translocation ability of SCPs outperforms conventional cationic-based CPPs both in payload delivery capacity and in reduced cell toxicity [12]–[15]. Thus, due to these outstanding characteristics, the design and/or production of this new class of intracellular delivery tools was considered a promising technology with particular high potential for nucleic acid delivery [17]. More recently, Liu and co-workers demonstrated that SCPs occur in nature, namely in humans [14]. However, the use of human proteins in drug delivery strategies may cause unwanted cellular responses to the stimulus due to systemic overload of an endogenous protein. The use of non-human SCPs as drug delivery scaffold would potentially minimize cellular responses.

Viruses are assemblies of nucleic acids and proteins, sometimes surrounded by a lipid bilayer. Viral capsid proteins, in particular, are optimized for interacting with nucleic acids. Thus, our hypothesis was that if they are “supercharged”, they might be able to penetrate cells carrying nucleic acids. To test this hypothesis, we examined the molecular weight and global net charge of the viral structural proteins (capsid - C, membrane - M and envelope - E) included in the ExPASy viral database Viralzone (http://viralzone.expasy.org). Our analysis showed that a fraction of these viral proteins, most of them capsid proteins, may indeed be included in the supercharged class of proteins. Dengue virus (DENV) capsid (C) protein is one of the most prominent examples of a protein with a high net positive charge combined with low molecular mass. Our groups have been working over the past years with DENV structural proteins, and more recently with the C protein [18]–[22] (Figure S1 in File S1).

DENV, a member of the *Flaviviridae* family, is an enveloped virus with a positive sense, single stranded genomic RNA (ssRNA) (∼11 kb), which forms a nucleocapsid with multiple C protein copies [23]. DENV C protein originally consists of 114 amino acid residues, which are reduced to 100 after release from the endoplasmic reticulum (ER) membrane. Its three-dimensional structure [24] shows the mature form to be a dimer with a global net charge of +42, i.e., a +1.97/kDa ratio (ProtParam [25]). Each monomer contains four α-helices (α1 to α4) connected by short loops. DENV C protein contain a conserved internal region proposed to interact with lipid membranes [24] and a sequence putatively responsible for viral RNA binding, characterized by an abundance of basic residues.

This work portrays DENV C protein as a member of the recently described class of SCPs. A comparative study of DENV C protein and two peptides derived from its conserved domains was also carried out. Recombinant DENV C protein and two synthetic peptides containing, respectively, its putative RNA-binding (pepR, residues 67–100) and membrane-binding (pepM, residues 45–72) domains, were studied to identify the domain responsible for the translocation capability of this SCP. Specifically, we assessed their ability to deliver short nucleic acid (ssDNA and siRNA) sequences by non-covalent binding, as observed for other chimeric viral CPPs [26]–[28]. The results suggest that naturally occurring SCPs may also serve as sequence templates for novel CPPs. Moreover, DENV C protein was itself a potent transfection agent transporting nucleic acid cargo into the intracellular space. This is probably a functional ability acquired by natural selection, which may have potential implications in DENV C protein role in the viral infection cycle. Given the close similarities among the capsid protein of DENV and those of other *Flaviviridae* family members such as West Nile (WNV), Yellow Fever (YFV) and tick-borne encephalitis (TBEV) viruses, our findings may be extrapolated to their respective capsid proteins.

## Materials and Methods

### Chemicals

The phospholipids 1-palmitoyl-2-oleoyl-*sn*-glycero-3-phosphocholine (POPC), 1-palmitoyl-2-oleoyl-*sn*-glycero-3-phosphoetanolamine (POPE), 1-palmitoyl-2-oleoyl-*sn*-glycero-3-phosphoserine (POPS) and 1-palmitoyl-2-oleoyl-*sn*-glycero-3-(phospho-*rac*-(1-glycerol)) (POPG) were purchased from Avanti Polar Lipids (Alabaster, AL, USA). Minimum essential medium α (α-MEM), Dulbecco’s modified Eagle’s medium (DMEM), fetal bovine serum (FBS), penicillin-streptomycin (Pen-Strep), phosphate buffer saline (PBS), ssDNA (ACG TGC TGA GCC TAC), ssDNA-Alexa488, and the fluorescent dyes Hoechst 33342 and Cell Mask Deep Red were purchased from Life Technologies (Carlsbad, CA, USA). Microscopy ibiTreat coated µ-slide 8 µ-well plates were purchased from Ibidi (Munich, Germany). HIV-1 Tat (47–57) was purchased from Bachem (Bubendorf, Switzerland). 4-(2-hydroxyethyl)-1-piperazineethanesulfonic acid (HEPES), citrate, NaCl and Triton X-100 were acquired from Sigma-Aldrich (St. Louis, MO, USA). Two buffers were used: 10 mM HEPES pH 7.4, and 10 mM citrate pH 5.5, both with 150 mM NaCl. Fmoc-protected amino acids were obtained from Senn Chemicals (Dielsdorf, Switzerland) and Fmoc-Rink-amide (MBHA) resin from Novabiochem (Merck, Darmstadt, Germany). 2-(1H-benzotriazol-1-yl)-1,1,3,3-tetramethyluronium hexafluorophosphate (HBTU) and N-hydroxybenzotriazole (HOBt) were from Matrix Innovation (Quebec, Canada). HPLC-grade acetonitrile, and peptide synthesis-grade N,N-dimethylformamide (DMF), dichloromethane (DCM), N,N-diisopropylethylamine (DIEA) and trifluoroacetic acid (TFA) were from Carlo Erba-SDS (Sabadell, Spain). All other reagents were of the highest quality available commercially.

### Computational Analysis of Viral Protein and Peptides

Global analysis of all structural viral proteins to identify potential hits for SCPs was performed using the Viralzone database from the ExPASy [25] consortium (http://viralzone.expasy.org) as source for all protein sequences in the viral families. Only fully characterized viruses, with carefully annotated protein localization and function, were considered in the analysis. Structural proteins were generally classified as envelope, membrane and capsid proteins according to their respective annotation. They were then submitted to ProtParam [25] in order to determine the protein global net charge to molecular weight (M_W_) ratio. This ratio was calculated for each protein and plotted versus the protein global net charge. In addition, viral protein sequences from which CPPs annotated at the CPPsite [29] had been derived were also analyzed and plotted. A threshold of +0.75/kDa was established according to the literature [2], [14] to identify positive hits for SCPs of viral origin.

The available tridimensional structure of DENV C protein (PDB ID: 1R6R) [24] was used. pepR and pepM tridimensional structure predictions were achieved by submitting their sequences to the I-TASSER web server [30] (http://zhanglab.ccmb.med.umich.edu/I-TASSER/I-Tasser). Secondary structure propensity of DENV C protein, pepR and pepM was obtained by submitting their amino acid sequences to the web server PSIPRED [31]. Helical wheel projections of both pepR and pepM were obtained by submitting their amino acid sequences to the online tool Heliquest [32] (http://heliquest.ipmc.cnrs.fr). All tridimensional representations of protein and peptides were done with the PyMol v1.4 molecular visualizer [33].

### pepR and pepM Synthesis

Both pepR (LKRWGTIKKSKAINVLRGFRKEIGRMLNILNRRRR – residues 67–100 of DENV-2 C protein) and pepM (KLFMALVAFLRFLTIPPTAGILKRWGTI – residues 45–72 of DENV-2 C protein), as well as their N-terminal fluorescein- or rhodamine B-labeled versions, were prepared by Fmoc solid phase peptide synthesis methods [34], purified by HPLC and characterized by MALDI-TOF mass spectrometry as previously described [35], [36]. Further details are given in the Supplementary Information S1 (Table S1 in File S1).

### DENV C Protein Expression and Purification

DENV C protein was expressed in *Escherichia coli* (strain Codon Plus) transformed with DENV-2 C protein gene (encoding residues 1–100), cloned into pET21a plasmid, and purified as described elsewhere [20], [21].

### Cell Culture

Baby hamster kidney (BHK-21), hepatocellular carcinoma (HepG2) and brain microvascular endothelial (BMEC) cell lineages were cultured in DMEM supplemented with 10% (v/v) FBS, 100 U.mL^−1^ penicillin-streptomycin, and placed at 37°C in a humidified atmosphere of 5% CO_2_. Primary astrocyte cultures were prepared [37] and maintained as described elsewhere [38]. Briefly, single cell suspensions obtained from carefully disrupted prefrontal cortex from newborn (1 to 2-day-old) mice brain were plated in culture dishes with DMEM supplemented with 20% (v/v) FBS and 10 U.mL^−1^ of penicillin-streptomycin and maintained at 37°C with 10% CO_2_. Cells were counted using a Fuchs-Rosenthal hemocytometer (Brand Wertheim). Cell viability was determined by the trypan blue dye exclusion method. Viability was always above 98%.

### Confocal Microscopy

An inverted confocal point-scanning Zeiss LSM 510 META microscope equipped with Diode 405–30, Argon2, DPSS 561–10, HeNe 594 and HeNe 633 lasers, and a temperature control incubator (37°C) with CO_2_ supply were used. Images were taken on µ-slide 8 µ-well plates with a Plan-Aprochromat 63× objective. Nucleus was stained with Hoechst 33342 at the concentration of 1 mg.mL^−1^. Briefly, BHK-21, HepG2 or astrocytes were plated at 1 to 4×10^4^ cells.mL^−1^ in Ibidi µ-slides and cultured for 2 days. For each sample, 1 µM ssDNA-Alexa488 was added, as well as 5 µM rhodamine B-labeled pepR or pepM, or 3 µM of unlabeled DENV C protein. The 4°C experiments were performed as previously described by Henriques et al. [39]. Cultured cells were maintained for at least 60 min at 4°C prior to ssDNA and DENV C protein addition. Images were recorded 15 min later. All images were analyzed by the image processor ImageJ v1.46 [40].

### Flow Cytometry

Flow cytometry experiments were performed using a FACScan from BD Biosciences (San Jose, CA, USA), with 3 detectors: FL1 (530/30 BP), FL2 (585/42 BP) and FL3 (>670 LP). A suspension of BHK-21 cells in a density of 2×10^6^ cells.mL^−1^ was prepared by trypsinization and incubated with 10 nM of ssDNA labeled with Alexa-488. FL1 and FL3 channels were recorded for 1 min before and 15 min after addition of 2 µM DENV C protein, pepM-rhodamine B, or Tat, or 4 µM pepR-rhodamine B in a FACScan (n = 3). BHK-21 cells transfected with the GFP-encoding plasmid were analyzed using FL1 channel for 1 min, to quantify the percentage of transfected cells, using DENV C protein, Lipofectamine or Tat as the transfection agent (n = 6).

For the kinetic acquisition mode, the FL1 and FL3 channels were recorded for 12 min immediately after addition of the previously complexed DENV C protein (2 µM) with 10 nM ssDNA-A488. Before the addition of the complex (t = 0), a 30 s acquisition of the cellular suspension was done for background correction in data analysis. After background subtraction, the average fluorescence signal of each channel was plotted *vs*. time.

### DENV C Protein-mediated pEGFP-C3 Transfection

BHK-21 cells were seeded at a 2×10^4^ cells.cm^−2^ density onto each dish of a 6-well plate. After 24 h, cells were transfected with 1 µg of the pEGFP-C3 plasmid using as the transfection agent either DENV C protein (2 µM), Tat (2 µM) or Lipofectamine 2000 (4 µL). Media were changed at t = 6 h. Positive transfection was followed after 48 h by cell fluorescence emission due to the expression of GFP, by microscopy or flow cytometry. For microscopy analysis, cells were harvested and seeded onto 8 µ-well plates and imaged in a confocal microscope. For flow cytometry analysis, cells were harvested by trypsinization and washed 3 times with PBS. FL1 fluorescence of the cytometer was recorded for 1 min to evaluate the percentage of GFP transfected cells (n = 6). Fluorescence excitation (400–500 nm) and emission (500–600 nm) spectra were further acquired in order to observe positive GFP signal in the transfected cells, as well as to conclude about cellular autofluorescence.

### DENV C Protein-mediated TLR3 Gene Silencing

TLR3 gene silencing assay was performed using BMEC. After 24 h of culture, BMEC were transiently transfected for silencing of TLR3 gene using small interfering RNA (siRNA; sc-36685 from Santa Cruz Biotechnology, Inc., Dallas, TX, USA). Transfection was performed with 0.06 µM of TLR3 siRNA using Lipofectamine 2000 (according to the manufacturer’s instruction), DENV C protein (3∶1 and 2∶1 protein:siRNA molar ratios), pepR or pepM (peptide:siRNA molar ratio of 5∶1), for 5 h. The silencing of TLR3 gene was analyzed by real-time PCR 48 h after transfection. Cells were harvested and lysed with TRIZOL reagent (Invitrogen, Inc., Carlsbad, CA, USA). Total RNA was treated with DNAse I (Fermentas, Thermo Fisher Scientific Inc., Pittsburgh, PA, USA) and first strand cDNA was synthesized using 2 µg of the extracted RNA using High-Capacity cDNA Archive Kit (Applied Biosystems, NY, USA) according to the manufacturer’s instructions. cDNA was diluted 1∶5 and 1 µl of each sample was used for real-time PCR (RT-PCR) using Power SYBR Green PCR master mix (Applied Biosystems, NY, USA). TLR3 gene identification was performed using the primers sense 5′ – AGTGCCCCCTTTGAACTCTT –3′, and antisense 5′ – ATGTTCCCAGACCCAATCCT –3′. GPDH gene was used as the housekeeping gene. The thermal cycler was used to monitor the SYBR Green signal at the end of each extension period for 40 cycles. The differences between the relative expression values for the TLR3 gene in the control and siRNA transfected samples were submitted to the two-tailed *t*-test to ascertain their statistical significance. The resulting data are the mean of three experiments with standard deviation.

### Preparation of Model Membranes

Large unilamellar vesicles (LUV), typically with 100 nm diameter, prepared by the extrusion protocol described elsewhere [41], [42], were used as biomembrane models. Briefly, lipid mixtures were prepared in rounded glass-flasks and dried in vacuum overnight. The solution was then rehydrated and submitted to 8 freeze/thaw cycles. Multilamellar vesicles (MLV) were collected at this stage, before performing the extrusion procedure with a 100 nm-pore membrane to obtain LUV.

### Lipid Membrane Translocation Assays

The intrinsic fluorescence intensity of pepR and pepM was acquired for 25 min, using an excitation wavelength of 280 nm and an emission wavelength of 350 nm. At *t* = 1 min, MLV or LUV (700 µM lipid) were added to a 15 µM solution of pepR or pepM, with or without 1 µM ssDNA. Upon insertion in the lipid bilayers, the Trp residue of the peptide experiences a hydrophobic environment and its fluorescence emission quantum yield increases, resulting in an increase of the fluorescence intensity. As MLV have lipid bilayers enclosed in other lipid bilayers, only a fraction of the lipid bilayer is exposed to interact with added peptide. In contrast, LUV are unilamellar, having all the lipid bilayer exposed to the added peptide. Proteins that translocate membranes will successively transpose the lipid bilayers of MLV until all the vesicles have inserted proteins. In this case, the final fluorescence intensity of the samples matches the final fluorescence intensity obtained with LUV. Non-translocating proteins only interact with the outer layer of MLV, so that they cannot reach the fluorescence intensity obtained with LUV. This method was described in detail elsewhere [43]. Fluorescence emission experiments were carried out with an Edinburgh Instruments (Livingston, UK) fluorescence Spectrophotometer model FS920, equipped with two double monochromators and a 750 W Xe lamp.

### LUV Fusion Assay

5 mM of POPC, POPC:POPG (4∶1), POPC:POPS (4∶1) or POPC:POPE (4∶1) LUV labeled with 1% NBD-POPE and 1% rhodamine B-POPE were prepared and mixed with the respective non-labeled LUV in a 1∶4 proportion. The vesicles suspension was then titrated with DENV C protein at concentrations up to 36 µM or with peptide:ssDNA conjugates at 4∶1 molar ratio with peptide (pepR and pepM) concentrations up to 36 µM. Fluorescence emission spectra were collected from 490 to 650 nm, with excitation at 470 nm and 10 nm bandpasses, after 15 min incubation with the protein or peptide. After the peptide addition and spectra acquisition, Triton X-100 1% (v/v) was added to establish the 100% vesicle fusion limit. Peptide fusion efficiency was determined by [44]:
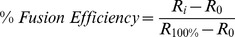
(1)where R_i_, R_0_ and R_100%_ are the ratios of the fluorescence intensity from the donor (NBD)/acceptor (rhodamine B) at the maximum emission wavelengths from each fluorophore at a given concentration of protein or peptide, at its absence, and after Triton X-100 addition, respectively [44]. Lipid fusion decreases FRET efficiency because the unlabeled and labeled vesicles fuse, diluting the lipids labeled with the dyes, increasing the average distance between donor and acceptor probes [44].

### Zeta-Potential Measurements

Zeta-potential (ζ-potential) measurements were performed in a Malvern Ζetasizer Nano ZS apparatus (Malvern, UK) with a backscattering detection at a constant 173° scattering angle, equipped with a He-Ne laser (λ = 632.8 nm). Ζetasizer folded capillary cells DTS 1060 (Malvern, UK) were used in the ζ-potential experiments. Aliquots of constant 200 µM of LUV were prepared to a final sample volume of 1 mL with the addition of pepM (0–100 µM), pepR (0–32 µM) or DENV C protein (0–6 µM) into a sterile eppendorf tube and then filtered into the ζ-potential cuvette. Filtered pH 7.4 HEPES buffer was used in all the samples. For each sample the instrument performed 20 scans (70 runs each), with an initial equilibration time of 15 min, at 25°C, and a constant voltage of 40 mV. Values of viscosity and refractive index were set at 0.8872 cP and 1.330, respectively. Data analysis was processed using the instrument Malvern’s DTS software to obtain the mean ζ-potential value.

## Results

### DENV C is a Supercharged Protein

**Figure 1:**
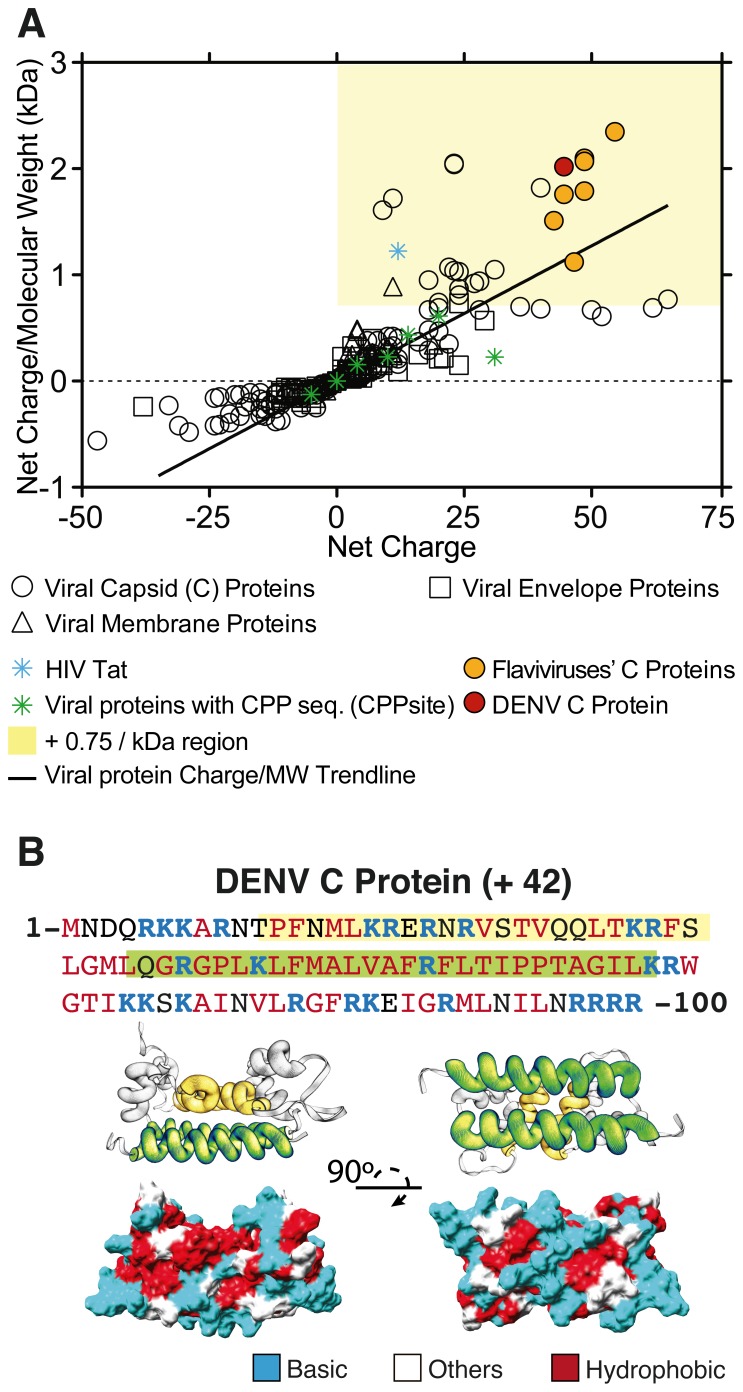
Viral structural proteins as supercharged proteins. **A)** Plot of formal net charge to Molecular Weight (M_W_) ratio *vs.* formal net charge of viral structural proteins (Capsid, Membrane and Envelope) annotated at Viralzone (http://viralzone.expasy.org). The yellow background region of the plot is the area with formal net charge/M_W_ exceeding +0.75/kDa, i.e. the region comprising the supercharged proteins [2], [12], [14]. A viral protein net charge/M_W_ trend line is plotted as a guide to the eye to facilitate the identification of the most significant supercharged proteins. Capsid proteins from flaviviruses (yellow solid circles) clearly form a subgroup of supercharged proteins. **B)** Linear sequence of DENV C protein monomer and structural representation of the dimer (PDB ID: 1R6R). Hydrophobic (MBS) and cationic (RBS) conserved regions are highlighted in yellow and green, respectively. The surface charge distribution of DENV C protein is represented: basic residues (blue), hydrophobic (red) and all other residues (grey) are shown. Tridimensional representation obtained by the PyMol software [33].

Structural proteins from each virus described in Viralzone [45] were annotated as membrane, envelope or capsid proteins and analyzed using previously described methods [2], [14] to identify naturally occurring supercharged human proteins. All proteins (270 total –160 capsid, 70 envelope and 40 membrane) were ranked by their formal global net charge/M_W_ and this parameter was plotted against formal net charge (Figure 1A). A protein with a net charge/M_W_ above +0.75 is considered to have intrinsic cell-penetrating tendency [2], [14]. Of the 270 viral proteins analyzed, 24 (8.9%) had net charge/M_W_ above +0.75. From those positive hits, 23 were viral capsid proteins (14.4% of total capsid proteins) and 1 was a membrane protein (2.5% of all membrane proteins). The *Flaviviridae* family was shown to be particularly rich in SCPs: all capsid proteins in this family (9) possess a charge/M_W_ higher than +1.07/kDa, the value obtained for hepatitis C virus core protein. YFV and DENV capsid proteins, with charge/M_W_ of +2.30/kDa and +1.97/kDa, respectively, showed the highest values (Figure 1A). It is noteworthy that viral proteins from which CPPs have been derived, such as Erns glycoprotein from classical swine fever virus, capsid protein from flock house virus, VP1 from Simian vacuolating virus 40 and k8 protein from human herpesvirus-8 [29], do not qualify as SCPs. The exception is the HIV trans-activator transcription (Tat) protein (Figure 1A), from which Tat, one of the most emblematic CPPs, is derived. These results show that the properties of isolated protein domains are not always reproduced in the whole protein structure.

Figure 1B shows DENV C protein amino acid sequence and charge distribution in the tridimensional structure of the dimer. All basic residues are significantly exposed to the solvent. Based on this charge distribution, it was suggested that the α4–α4’ region, rich in cationic residues, could bind nucleic acids, whereas the more hydrophobic region, located at the α2–α2’ helices, could interact with lipid membranes [24]. This 3D amphipathicity suggests the existence of domains with specific nucleic acid- and cell membrane-binding functionalities (Figure S1A in File S1).

### DENV C Protein Delivers Nucleic Acids into Mammalian Cells

**Figure 2:**
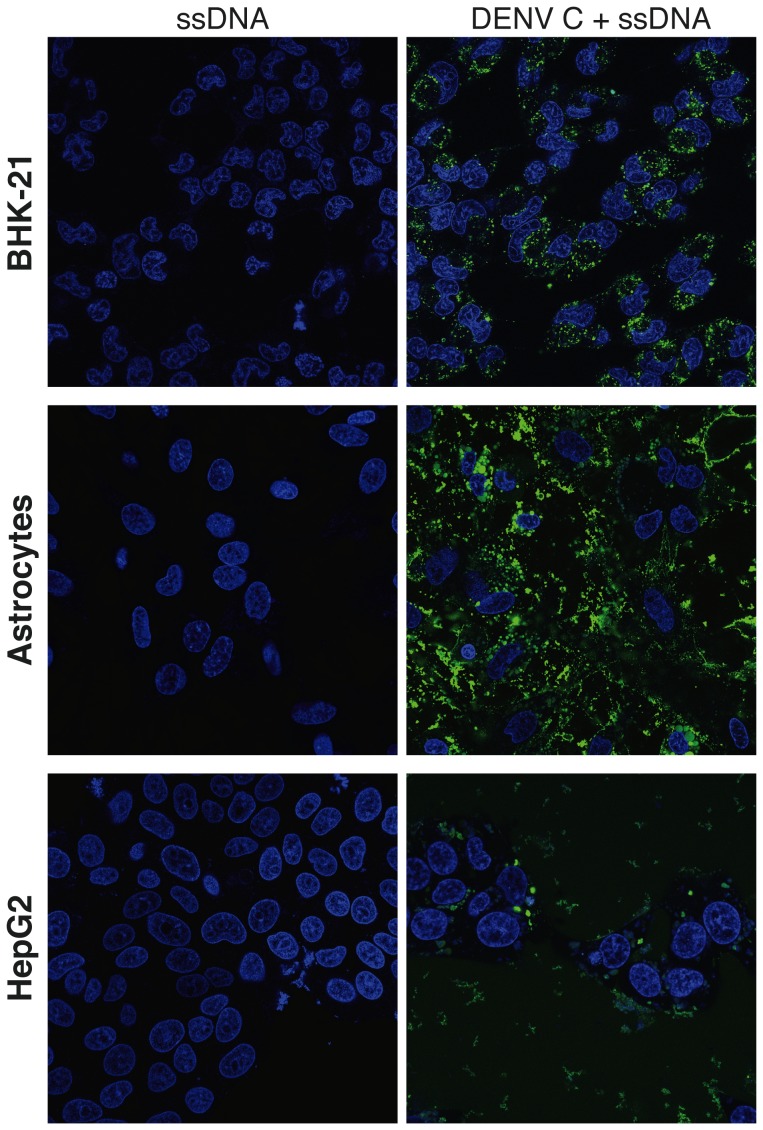
DENV C protein-mediated delivery of ssDNA into different cells. Confocal imaging of BHK-21 cells, astrocytes and HepG2 cells. Cells were cultured for two days and then stained with Hoechst 33342 at 1 mg.mL^−1^ (blue – nuclei), followed by the addition of ssDNA-Alexa488 at 1 µM (green) and unlabeled DENV C protein (3 µM).

DENV C protein is a lipid- and nucleic acid-interactive protein. To test the hypothesis that it mediates the transport of nucleic acids across lipid membranes, as expected for a supercharged protein, BHK-21 cells, HepG2 cells and astrocytes were imaged by confocal microscopy after incubation with ssDNA-Alexa488 and DENV C protein (Figure 2). Delivery of ssDNA by DENV C protein was detected in all cell models, showing that this protein delivers cargo to the cell interior regardless of cell type. Higher fluorescence intensity and more diffused emission are observed in astrocytes relative to the other cells. Interestingly, brain cell plasma membranes are rich in anionic lipids [46], [47], therefore facilitating interaction with cationic proteins such as DENV C.

It should be stressed that in the experimental conditions, DENV C protein and its derived peptides are not cytotoxic regardless of cell type. Cell viability assays were carried out imaging BHK-21 cells and PBMC with the marker TO-PRO3 (Figure S2 and Figure S3, respectively in File S1).

**Figure 3:**
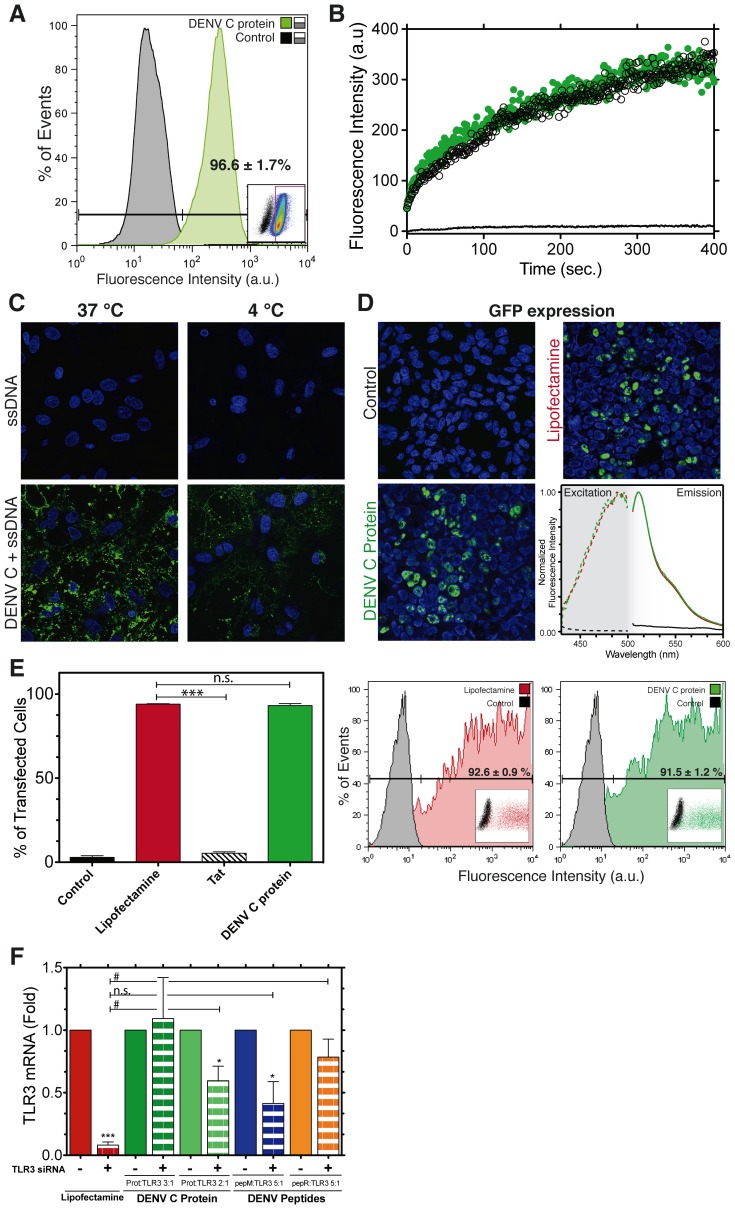
Quantification of DENV C protein-mediated translocation of ssDNA, GFP-encoding plasmid and TLR3 siRNA into cells. **A)** Flow cytometry fluorescence distribution histograms of trypsinized BHK-21 cells incubated with ssDNA-Alexa488 in the presence or in the absence (control) of DENV C protein. The percentage of BHK-21 cells positive for DENV C protein-mediated ssDNA internalization is indicated. **B)** Real time-flow cytometry of DENV C-mediated ssDNA intracellular delivery in BHK-21 cells at 4°C (black) or 37°C (green). The average fluorescence intensity signal of ssDNA is plotted over time. **C)** DENV C-mediated ssDNA delivery was also carried out on astrocytes at 37°C and 4°C, and imaged by confocal microscopy. Nuclear staining with Hoechst 33342 at 1 mg.mL^−1^– blue. **D)** Confocal imaging of BHK-21 cells cultured for one day and then transfected with pEGFP-C3 using DENV C protein or Lipofectamine (control – no transfecting agent). Positive GFP expression (green) was imaged 48 h after transfection. Nuclear staining with Hoechst 33342 at 1 mg.mL^−1^– blue, as in C). The right panel shows GFP fluorescence excitation (dashed lines) and emission (solid lines) spectra in BHK-21cells suspensions after Lipofectamine- (red) and DENV C-mediated (green) transfections (control – black). **E)** Percentage of GFP-positive cells, quantified by flow cytometry after transfection with Lipofectamine (red), Tat (black and white) or DENV C protein (green). Flow cytometry histograms of BHK-21 cells after transfection with Lipofectamine (red) or DENV C protein (green) are shown on the right. The fraction of GFP-positive cells is indicated. Statistical analysis was carried out using the two-tailed *t-*test (n.s. – non-significant; *** – p<0.0003)**. F)** TLR3 gene silencing assay using siRNA sc-36685 in BMEC. Transfection was performed using Lipofectamine (red), DENV C protein (green), pepM (blue) or pepR (orange). TLR3 gene silencing was analyzed by real-time PCR 48 h after transfection. Results reflect TLR3 expression changes in the presence and absence of the TLR3 siRNA for each transfection agent tested. Relative expression values *vs.* control (absence of siRNA) were analyzed by the two-tailed *t*-test (***– p<0.0007; * – p<0.025). Relative expression values *vs*. Lipofectamine were also analyzed (n.s. – non-significant; # – p<0.04). The resulting data are the mean of three experiments with standard deviation.

BHK-21 cells were selected for further studies on the delivery mechanism using flow cytometry (Figure 3A,B). After adding the DENV C protein:ssDNA mixture to a BHK-21 cells suspension, 96.6±1.7% of the cells internalized the ssDNA (Figure 3A). In addition, Figure 3B shows that the internalization of ssDNA is very fast (400 s to completion) and the kinetics does not vary between 37°C and 4°C, indicating that this SCP is able to translocate cellular membranes directly, without the use of endocytic machinery. Figure 3C also shows intracellular delivery of the ssDNA molecule at both temperatures in astrocytes. Therefore, DENV C protein is able to cross biological membranes with no need for permeabilization agents and regardless of cell-specific receptors or endocytic routes.

DENV C protein-mediated nucleic acid delivery was also tested in BHK-21 cells, with functional nucleotide sequences, namely a GFP-encoding plasmid (pGFP), and in BMEC with small interfering RNA (siRNA) targeting the Toll-like receptor 3 (TLR3) gene (Figure 3D,E,F). DENV C protein translocated pGFP into BHK-21 cells with full preservation of functionality: GFP, identified by its fingerprint spectra in Figure 3D, was expressed with an efficiency similar to the observed using Lipofectamine as transfecting agent (Figure 3D and E). In addition, DENV C protein outperformed Tat, a classic HIV-derived CPP [48], which achieved much lower transfection levels, showing that the functional translocation of nucleic acids achieved by DENV C protein is not a spurious effect. Furthermore, TLR3 siRNA transfection was achieved using DENV C protein as transfecting agent (Figure 3F), although not as effectively as Lipofectamine. Altogether, results from Figures 2 and 3 demonstrate that DENV C protein is a SCP that efficiently delivers functional nucleic acids into mammalian cells.

It is worth stressing that DENV C was as efficient as Lipofectamine for pGFP transfection but not for siRNA transfection. It is frequent among cell penetrating peptides to have the efficiency of delivery dependent on the cargo [49], [50]. Our result shows that DENV C is particularly fit to transport large genomes, which raises the hypothesis that DENV C protein may participate in viral genome transduction in nature.

### Dissecting DENV C Protein-mediated Membrane Translocation

**Figure 4:**
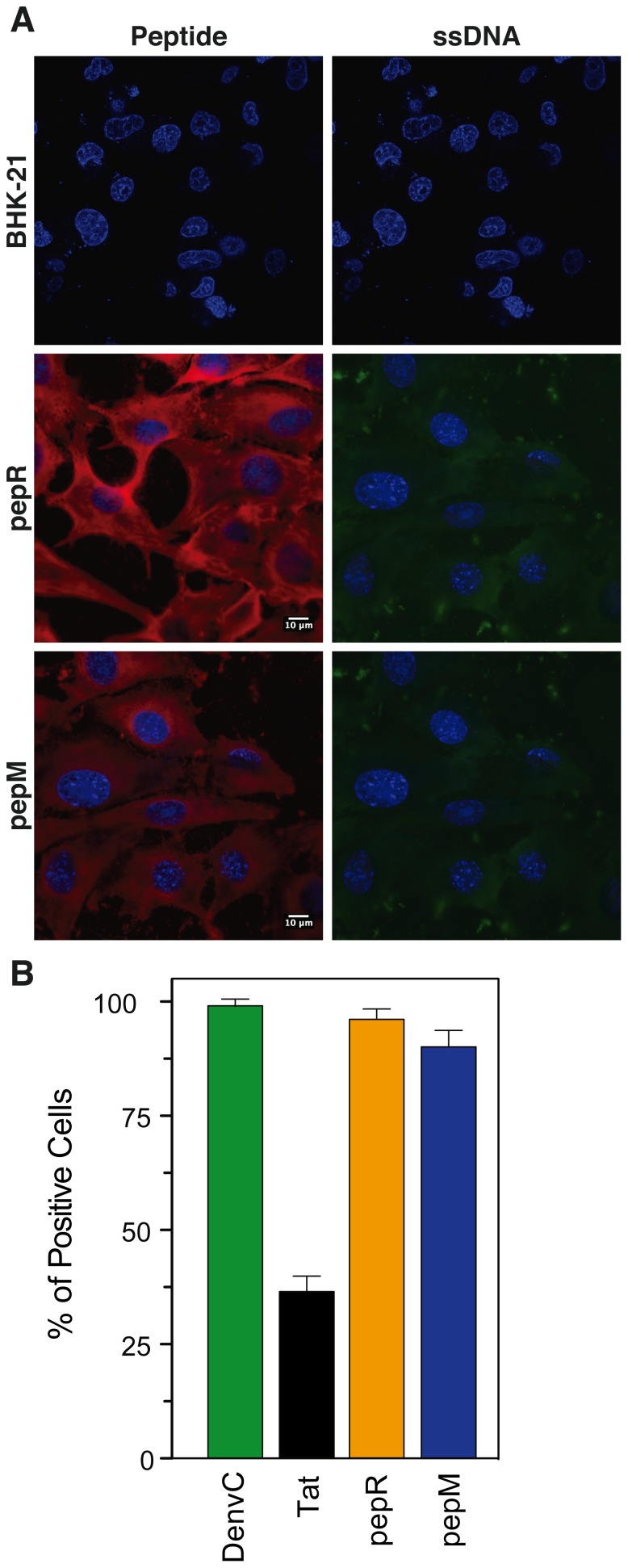
Cell delivery of ssDNA mediated by DENV C protein-derived peptides (pepR and pepM). **A)** Confocal imaging of BHK-21 cells. Cells were cultured for two days and then stained with Hoechst 33342 at 1 mg.mL^−1^ (blue – nuclei), followed by the addition of 5 µM of either pepR-rhodamineB or pepM-rhodamineB (red) conjugated with 1 µM of ssDNA-Alexa488. **B)** Flow cytometry quantification of the cellular internalization of ssDNA labeled with Alexa488, by using pepM, pepR, DENV C protein or Tat as delivery vectors, in BHK-21 cells.

**Figure 5:**
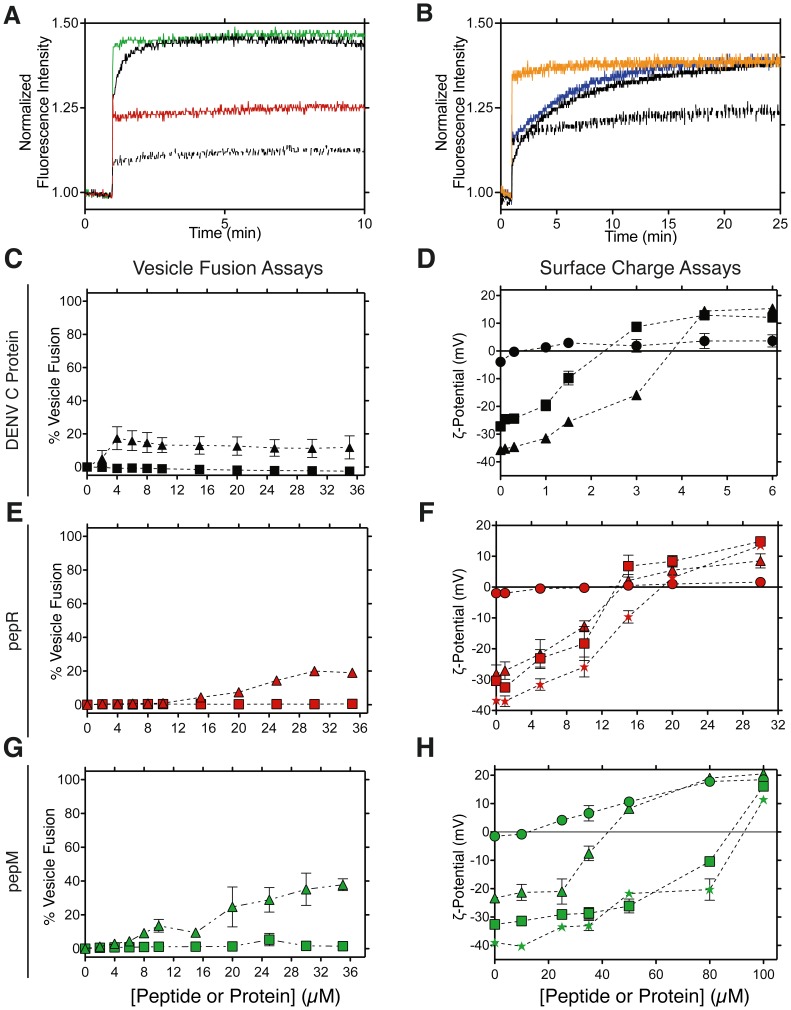
DENV C protein and DENV C protein-derived peptides pepR and pepM interaction with lipid vesicles. **A)** Lipid membrane translocation of pepR (red; dashed black) and pepM (green; solid black) in lipid vesicles of POPC:POPG 4∶1 (MLV – black; LUV – colored). **B)** Lipid membrane translocation of pepR-ssDNA (orange; dashed black) or pepM-ssDNA (blue; solid black) in lipid vesicles of POPC:POPG 4∶1 (MLV – black; LUV – colored). In **C, E, G)** LUV composed of POPC:POPG 4∶1 (triangles), or POPC:POPE 4∶1 (squares) were titrated with C) DENV C protein (black), E) pepR:ssDNA or G) pepM-ssDNA complexes (red and green, respectively) up to 36 µM, with a 4∶1 peptide:ssDNA molar ratio, at pH 7.4. The percentage of vesicle fusion dependence on concentration is represented. In **D, F, H)** ζ-potential of LUV composed of POPC (circles) or POPC:POPG (4∶1- triangles; 3∶2 - squares; 2∶3 - stars) titrated with D) DENV C protein, F) pepR or G) pepM up to 6, 32 and 100 µM, respectively, at pH 7.4. ζ = 0 is the point of vesicle surface electroneutrality.

To gain insight on the molecular basis of the remarkable functionality of DENV C protein as a transmembrane carrier, we studied the membrane-translocating properties of two isolated domains (pepR and pepM – Figure S1B and Figure S1C in File), using cells (Figure 4) and lipid vesicles (Figure 5A,B). Both peptides suffice to enter into cells (Figure 4A) and deliver ssDNA (Figure 4A,B); indeed, the percentage of ssDNA-positive cells using pepR and pepM for translocation was similar to that obtained with the whole DENV C protein (Figure 3A *vs.* Figure 4B). To better ascertain the efficacy of peptide- *vs.* protein-mediated delivery of functional cargoes, TLR3 siRNA transfection was also performed using pepR and pepM as delivery agents (Figure 3F). In this case, only pepM was able to deliver functional short nucleic acids into cells, but using a higher vector:cargo molar ratio relative to DENV C protein (5∶1 *vs.* 2∶1, respectively), supporting the hypothesis that SCPs are indeed more potent vectors than CPPs.

Interestingly, the staining in Figure 4A shows differences between pepR and pepM. The former appears as aggregates close to the nucleus, while the latter seems more diffuse. pepR contains a nuclear localization sequence (NLS) [51], which explains the location near the nucleus. The diffuse spreading of pepM in the cytoplasm, at variance with pepR, suggests that the latter enters cells through an endocytic route whereas pepM translocates the plasma membrane directly. This hypothesis also explains why in the experiments depicted in Figure 3F, pepR did not transfect TLR3 siRNA efficiently, but it was as efficient as pepM for transfection of ssDNA, as shown in Figure 4B. A further exploration of the translocation capabilities of both pepR and pepM, with or without cargo (ssDNA), was done on lipid vesicles of different composition (POPC and POPC:POPG (4∶1)) using a previously developed methodology [43] (Figure 5 and Figure S5 in File S1). Figure 5A shows that pepM is able to translocate artificial lipid bilayers in which anionic lipids are present; in contrast pepR is not able to translocate membranes regardless of lipid composition or cargo (Figures 5A and 5B, respectively). This suggests that pepM translocates bilayers in a receptor- or transporter-independent way, in tune with the ability of DENV C protein to deliver ssDNA at 4°C (Figures 3B,C). The divergent behavior of pepR and pepM suggests that the cationic domain favors non-covalent nucleic acid binding [26], [27], while the hydrophobic one may favors membrane interaction, perturbation and consequent translocation. It should be noted, however, that these domains cannot be simplistically assigned to the nucleic acid-binding and membrane-binding functionalities of DENV C protein, respectively, since both peptides have the ability to complex ssDNA and to interact with lipids [36], [52].

### DENV C Protein and Lipid Membrane Fusion

Vesicle fusion induced by DENV C protein, as well as the pepR:ssDNA and pepM:ssDNA complexes was studied using LUV simultaneously doped with labeled lipids forming the NDB-rhodamine B FRET pair. Figure 5C,E,G shows that pepR:ssDNA and pepM:ssDNA complexes, despite their high partition to membranes (Figure S4 and Table S2 in File S1), have moderate ability to promote fusion of LUV rich in anionic phospholipids. Concomitant to membrane fusion, the peptides neutralize the surface of the vesicles as revealed by ζ-potential measurements (Figure 5D,F,H). DENV C protein alone is unable to induce extensive membrane fusion, but despite its low fusogenic activity, the extent of the interaction with membrane is high: lipid neutralization is achieved at low protein concentrations (Figure 5D), suggesting a transient pore or the carpet model as the translocation mechanism [7], [10].

## Discussion

Can supercharged proteins be a result of protein natural evolution towards the acquisition of specific functions? Or are they simply peculiar proteins with minor biological significance? While it is reasonable to accept that capsid proteins possess a higher net positive charge than other viral structural proteins, as they need to interact with a negatively-charged viral genome, is there a reason to expect them also to efficiently translocate lipid membranes? In principle, and in addition to interacting with the viral genome and other capsid protein copies to form the nucleocapsid, capsid proteins can also be expected to facilitate the access and traffic of the viral genome within the host cell. Thus, it is not unreasonable to assume that these proteins evolved to become multitask proteins. In simple systems, evolution towards the acquisition of multifunctional features is a powerful driving force. In *E. coli*, for instance, 100 out of 607 known enzymes are multifunctional [53]. The high net charge/M_W_ of viral capsid proteins might be the outcome not only of the selective pressure towards the acquisition of nucleic acid-packing properties, but also illustrates the evolutionary convergence of proteins sharing the common essential feature of translocating cell membranes. More specifically, the above-demonstrated ability of DENV C protein to facilitate the translocation of a large nucleic acid molecule across cell membranes suggests that during natural evolution it acquired an additional role in the virus life cycle: genome translocation. During maturation from early to late endosome, the lipid composition of the endosomal membrane increases in anionic character [54], an event already proposed as determinant for DENV E protein-mediated endosome-viral envelope fusion [55]. It has been argued, however, that this change in lipid composition may not be sufficient for optimal membrane fusion to occur, as the process is also dependent on the mixture of lipid stalks, hemifusion of lipid bilayers and pore formation [56], [57]. Based on the experimental evidence gathered in this work, it is reasonable to hypothesize that DENV C protein helps E protein during fusion. After viral endocytosis, DENV E protein (in the trimeric conformation) mediates the attachment of viral to endosome membrane, promoting hemifusion [56], [58], [59]. We propose that, in subsequent steps, DENV C protein, due to its membrane translocation ability, helps to deliver ssRNA into the cytosol. Also, our work suggests the need to revisit the role of the capsid proteins from flaviviruses in the infection cycle. Some concerns about the current view of the viral fusion phenomenon have been already raised [57], [59], [60], and our results support a reappraisal of the possible functionalities of capsid proteins along the viral life cycle. Pierson and Kielian [59], for instance, stated very recently that, despite the huge and clear advances on the study of DENV organization at a structural and molecular level during the infection cycle steps, the description of this process is still incomplete for stages after hemifusion. Our findings suggest that DENV C cooperates with DENV E when viral envelope and endosomal membranes meet, and that this synergism is relevant in order to successfully infect the host cell.

Additionally, it is an attractive hypothesis that DENV C protein may be responsible for cell-to-cell direct viral transmission, eluding viral release, as already shown for HIV infections [61], [62], for instance. As DENV C protein suffices to translocate cell membranes carrying nucleic acids, DENV cell-to-cell infection promoted by C protein is also a strong possibility.

### Concluding Remarks

Supercharged proteins are more than huge cationic CPPs. This class of proteins is able to enter into mammalian cells using different pathways and exhibit cell-penetration and macromolecule-delivery capabilities that outperform well studied CPPs, mainly due to differences in charge density, structure, or surface area [2], [14], [15]. Previous work on this subject, either by mutagenic engineering [12], [13] or direct identification in the human proteome [2], [14], has portrayed SCPs as rising stars of a novel drug delivery paradigm, with significant potential in biomedicine [17]. We now show that a certain family of viruses, the *Flaviviridae*, uses SCPs as capsid proteins. Spatial and structural constraints [63] may have driven capsid proteins towards multifunctionality, hence to become SCPs with the dual ability to bind nucleic acids and translocate membranes.

Our specific finding of DENV C protein as a SCP with a strong ability to deliver nucleic acids is relevant not only by the intriguing possibility that translocating the virus genome during viral fusion is a main biological function of flaviviruses’ capsid proteins, but also by its potential biomedical applications. Most drug delivery formulations rely on cationic compounds. CPPs, biopolymers, nanoparticles and liposomes are examples of systems that are able to penetrate mammalian cells [64]. CPPs are particularly promising due to their low toxicity and synthetic versatility. While several well-known CPPs derive from viral protein sequences [29], having intrinsic CPP sequences is not enough to make the parent protein a SCP (Figure 1A). Thus, though we have successfully designed pepR and pepM as intrinsic CPPs within the DENV C protein sequence and proved their ability to deliver nucleic acid cargo into cells, DENV C protein outdoes both peptides in effectiveness, as found previously for other proteins [15], while keeping low toxicity (Figure S2 and Figure S3 in File S1). This would set aside SCPs as an independent class of proteins/delivery systems, distinct from CPPs.

## Supporting Information

File S1 *Section 1:* DENV C protein structural information, as well as pepR and pepM design and synthesis – Figure S1 and Table S1. *Section 2:* Additional confocal microscopy and TO-PRO3 cellular viability assays – Figure S2 and Figure S3. *Section 3:* Supplementary methods and results on model studies with lipid membranes (membrane partition, lipid membrane fusion and zeta-potential experiments) and FRET assay – Figure S4, Figure S5 and Table S2.
(DOCX)
