# Supplementary material for: Intracellular Nucleic Acid Delivery by the Supercharged Dengue Virus Capsid Protein
Source: PLoS One. 2013 Dec 5;8(12):e81450. doi: 10.1371/journal.pone.0081450 (PMC3855322; doi:10.1371/journal.pone.0081450)
Supplement: File S1 — Section 1: DENV C protein structural information, as well as pepR and pepM design and synthesis – Figure S1 and Table S1. Section 2: Additional confocal microscopy and TO-PRO3 cellular viability assays – Figure S2 and Figure S3. Section 3: Supplementary methods and results on model studies with lipid membranes (membrane partition, lipid membrane fusion and zeta-potential experiments) and FRET assay – Figure S4, Figure S5 and Table S2. (DOCX) [file pone.0081450.s001.docx]

**SUPPLEMENTARY MATERIAL**

**Intracellular Nucleic Acid Delivery by the Supercharged Dengue Virus Capsid Protein**

João Miguel Freire, Ana Salomé Veiga, Thaís M. Conceição, Wioleta Kowalczyk, Ronaldo Mohana-Borges, David Andreu, Nuno C. Santos, Andrea T. Da Poian and Miguel A. R. B. Castanho

Table of Contents

S1: DENV C protein information 2

Identification of translocation domains in DENV C protein - pepR and pepM 2

− pepR and pepM chemical synthesis 4

S2: Confocal microscopy 5

PBMC and BHK cell viability assays 5

S3: Studies on model membranes 8

Lipid membrane partition 8

Membrane translocation assay 10

References 12

# S1: DENV C protein information

## Identification of translocation domains in DENV C protein - pepR and pepM

Dengue virus (DENV) capsid (C) protein contains two conserved internal regions: one hydrophobic and the other highly cationic [1,2]. One can observe that the charged region is located at one edge of the protein and the hydrophobic at another, which in the tridimensional structure of DENV C protein results in the protein being amphipathic (Figure S1A). C protein sequence consists of 114 amino acid residues, being reduced to 100 residues after the release from the endoplasmic reticulum (ER) membrane. It has 26 basic amino acid residues and only two acidic residues. The mature DENV C protein forms dimers with a global net charge of +42 (ProtParam [3]), each monomer containing four α-helices (α1 to α4) connected by short loops. Based on this charge distribution, the α4-α4’ region, localized at the C-terminal, has been proposed to bind the viral RNA [2]. Likewise, the hydrophobic core located at the α2-α2’ region would be responsible to interact with lipid membranes [2]. Therefore, two distinct domains, each with a specified function, were assigned to DENV C protein: an RNA-binding domain (RBD), responsible for viral RNA binding, and a membrane-binding domain (MBD), proposed to interact with lipid membranes [2] (Figure S1A).

We designed and synthesized two peptides containing the putative RBD and MBD of DENV C protein, respectively, pepR (LKRWGTIKKSKAINVLRGFRKEIGRMLNILNRRRR – residues 67-100 of DENV-2 C protein) and pepM (KLFMALVAFLRFLTIPPTAGILKRWGTI – residues 45-72) (Figure S1B). Both peptides have a tryptophan residue, which makes them intrinsically fluorescent. pepR has 35 amino acid residues and a net formal charge of +12 at physiological pH (Figure S1B). The corresponding domain in the protein forms an amphipathic α-helix, having the positively charged residues at one side of the α-helix and the hydrophobic residues at the other side. pepM has 28 amino acid residues and a formal net charge of +4 at physiological pH 7.4 (Figure S1B). It is essentially hydrophobic, with the charged residues dispersed in the sequence. This approach, using peptides to model proteins [4], has already been applied with success on HIV fusion protein [5], for instance.


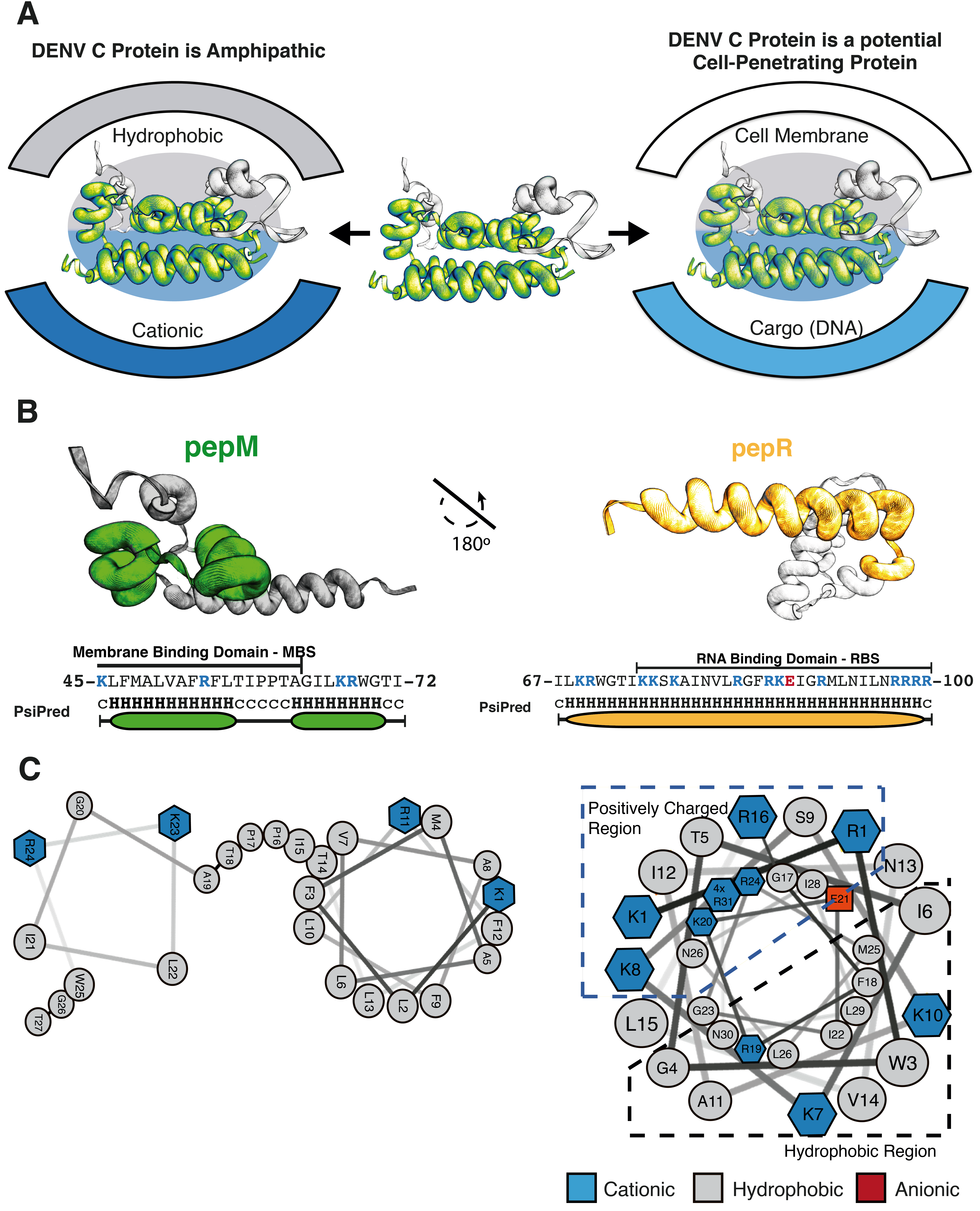


**Figure S1: DENV C protein sequence details. A)** DENV C protein is amphipathic, which is common in membrane-active peptides and proteins. **B)** DENV C protein membrane- and RNA- binding domains (PDB ID: 1R6R) and pepM (green) and pepR (yellow) localization within DENV C protein structure. **C)** Schematic representation of pepM (left) and pepR (right) as Henderson helical wheel projection obtained with the software Heliquest (http://heliquest.ipmc.cnrs.fr). The three-dimensional structure prediction was obtained at the web server I-TASSER [6]. The conformational representations were carried out with the PyMol software [7]. Positively charged residues (blue), hydrophobic and non-charged residues (grey) and negatively charged residues (red) are highlighted.

### pepR and pepM chemical synthesis

Both pepR and pepM, as well as their N-terminal carboxyfluorescein (CF)- or Rhodamine B (RhB)-labelled versions (Table S1), were prepared by solid phase synthesis methods. Automated syntheses was performed in an ABI433 peptide synthesizer (Applied Biosystems) running standard Fmoc (FastMoc) protocols [8] at 0.1 mmol scale on Fmoc-Rink-amide MBHA resin. Eightfold excess of Fmoc-L-amino acids and HBTU/HOBt, in the presence of double that molar amount of DIEA, was used for the coupling steps, with DMF as solvent. All side-chain functions were protected with TFA labile groups. Unlabeled pepR and pepM resins were N-deblocked with piperidine/DMF (20% v/v) prior to full deprotection and cleavage with TFA/H_2_O/TIS (95:2.5:2.5 v/v, 90 min, RT). For CF or RhB labeled versions, N-deblocked resin was placed in a synthesis syringe and treated with a 10-fold excess of dye and DIPCDI in DMF, then deprotected and cleaved as above. Peptides were isolated from the cleavage mixture by ether precipitation, redissolved in 10% HOAc, lyophilized and purified to >95% homogeneity by reverse phase HPLC. Their identities were confirmed by MALDI-TOF mass spectrometry.

**Table S1. Sequences and molecular weights of pepM, pepR and their fluorescent derivatives**

|  | **Sequence** | **Formula** | **MW [Da]** | **[M+H^+^]** |
| --- | --- | --- | --- | --- |
| pepR | LKRWGTIKKSKAINVLRGFRKEIGRMLNILNRRRR | C_189_H_335_N_69_O_42_S_1_ | 4278.2 | 4276.4 |
| CF-pepR | CF-LKRWGTIKKSKAINVLRGFRKEIGRMLNILNRRRR | C_210_H_345_N_69_O_48_S_1_ | 4636.6 | 4635.1 |
| RhB-pepR | RhoB-LKRWGTIKKSKAINVLRGFRKEIGRMLNILNRRRR | C_217_H_364_N_71_O_44_S_1_ | 4703.4 | 4704.5 |
| pepM | KLFMALVAFLRFLTIPPTAGILKRWGTI | C_155_H_250_N_38_O_31_S_1_ | 3173.9 | 3173.0 |
| CF-pepM | CF-KLFMALVAFLRFLTIPPTAGILKRWGTI | C_176_H_260_N_38_O_37_S_1_ | 3532.3 | 3532.1 |
| RhB-pepM | RhoB-KLFMALVAFLRFLTIPPTAGILKRWGTI | C_183_H_279_N_40_O_33_S_1_ | 3599.2 | 3597.2 |

# S2: Confocal microscopy

## PBMC and BHK cell viability assays

Potential biasing of the cell imaging results may occur if the peptides or ssDNA severely perturb cell homeostasis, particularly membrane integrity. Molecular probes sensing cell viability have been used in order to perform toxicity tests of a wide range of compounds and organisms [9]. We tested cell viability at the experimental conditions using the cell viability marker TO-PRO3. This molecule is a membrane integrity indicator, which binds to double strand DNA (dsDNA) molecules [9]. An intact cell membrane is impermeable to the fluorescent dye and no nuclear labeling by TO-PRO3 occurs (Figures S2 and S3). When the cells are imaged in buffer at 37ºC, the cell viability dye does not stain almost any cell, indicating that the cells in the panel are viable (Figure S2). Neither peptides nor DENV C protein (up to 5 µM) showed toxic effects. A positive control for cell death was performed with the addition of methanol 25% (v/v) to each well (methanol is known to be toxic to human tissues [10]). Almost all cells were stained by TO-PRO3 in the methanol panels, indicating membrane integrity disruption (Figure S2). Three hours after the methanol treatment, BHK cells secrete small vesicles, indicative of an apoptotic stage.

Significant toxicity levels in PBMC experiments were observed with the addition of 5 µM pepR (Figure S3). Cell incubation with DENV C protein at a concentration of 5 µM led to an increased TO-PRO3 staining when compared to the buffer control; however, it did not show the same severity of damages as the addition of the same amount of pepR. In the DENV C protein experiments, partial staining was observed at the cell membrane level, but there was no staining of the PBMC nucleus, such as on the methanol or pepR 5 µM assays. When pepR 3 µM was added, similar results were obtained: no nucleus staining is observed, but there is a certain degree of unspecific staining at the cell membrane level. pepM did not show any evidence of inducing cellular toxicity.


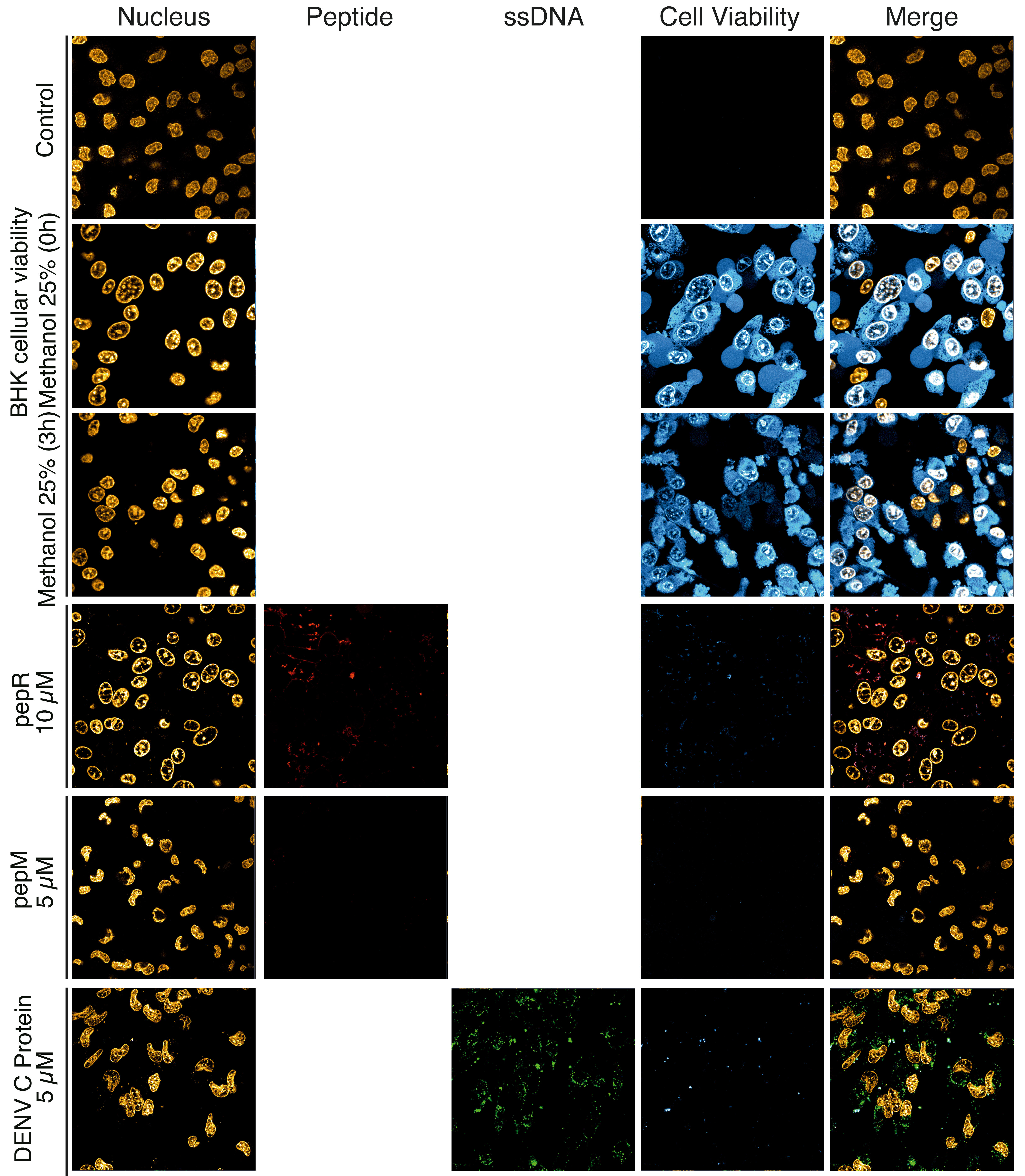


**Figure S2:** **Confocal imaging** **BHK cells: viability assays.** BHK cells were stained with Hoechst 33342 at 5 µg.mL^-1^ (nucleus – yellow) and cell viability marker TO-PRO3 (cyan) at 1 µg.mL^-1^, and then titrated with pepR or pepM labeled with Rhodamine B (red), or with DENV C protein (unlabeled). A positive control of cellular death was performed using methanol 25% (v/v). DENV C protein cellular translocation was detected by the appearance of ssDNA inside the cells. The increase in nuclear staining from TO-PRO3, due to dsDNA binding, is indicative of membrane disruption and cell death.

The interaction of the charged region from DENV C protein (pepR) with the nucleus may contribute to the severity of DENV-associated pathologies. PepR has already been described as a potential antimicrobial peptide (AMP) candidate [11], with minimum inhibitory concentration for some bacteria similar to other molecules of this class. In addition, pepR localization around the nucleus (possibly due to presence of NLS [4] – Figure S1A) may induce PBMC apoptotic behavior. Indeed, nuclear localization of DENV C protein has already been proven to be required for DENV to induce cell apoptosis [12]. The interaction of the highly charged domain of DENV C protein included in pepR with the nuclear membrane may be one of the molecular events underlying DENV-associated clinical conditions. In Dengue hemorrhagic fever, for instance, blood cells are severely affected (leading to thrombocytopenia and leukopenia) [13]. This observation is in agreement with the dual AMP and cell-penetrating peptide (CPP) properties of molecules such as pepR (lysine and arginine-rich amphipathic peptide), which strengthens the fact that both molecular categories are far from being distinct from each other [14,15].


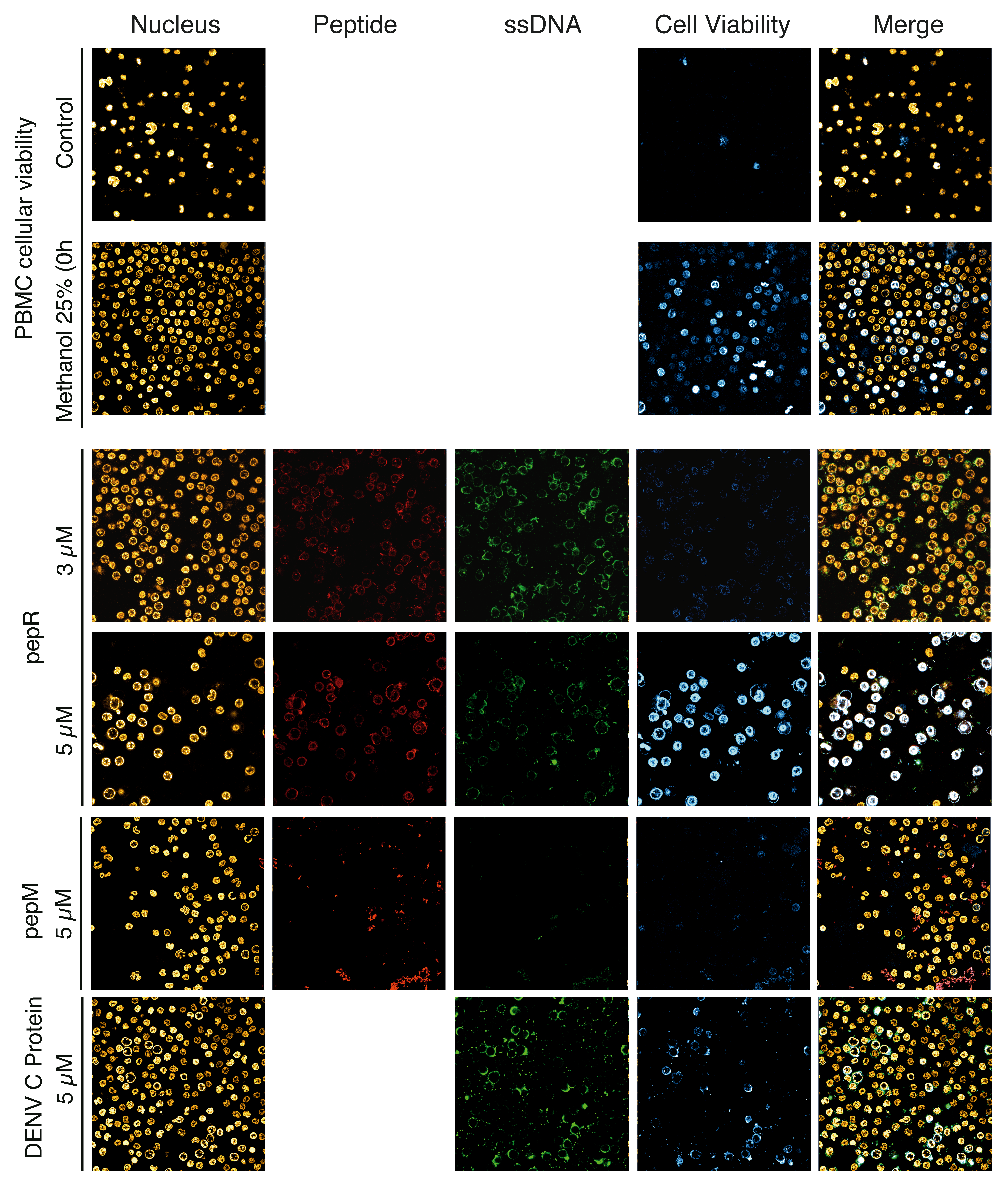


**Figure S3:** **Confocal imaging** **PBMC: viability assays.** PBMC were stained with Hoechst 33342 at 5 µg.mL^-1^ (nucleus – yellow), and cell viability marker TO-PRO3 (cyan) at 1 µg.mL^-1^, followed by the addition of pepR or pepM labeled with Rhodamine B (red), or with DENV C protein (unlabeled) at 5 μM final concentration. A positive control of cellular death was performed by the addition of methanol 25% (v/v). DENV C protein cellular translocation was detected by the appearance of ssDNA inside the cells, at variance with controls. The increase in nuclear staining by TO-PRO3, due to dsDNA binding, is indicative of membrane disruption and cell death.

# S3: Studies on model membranes

## Lipid membrane partition

The extent of membrane partition of peptides having fluorescent amino acid residues can be achieved by monitoring the intrinsic fluorescence emission. However, in several cases, peptides are not intrinsically fluorescent and the derivatization with a suitable fluorophore may be a valid approach to quantify their membrane interaction, assuming that this modification does not alter significantly the structural and functional properties of the peptide. In our case, the use of labeled peptide was not due to lack of intrinsic peptide fluorescence, but due to mathematical simplicity and fitting (for detailed explanation see Freire et al. [16]).

The definition and quantification of a partition constant of a solute between an aqueous and a lipid phase, *K_p_*, is already described elsewhere [17-19]. Briefly, for a chosen spectroscopic signal, there is a balance between the signals from the molecules located in each phase, which relies on the fractional distribution of the molecules between aqueous and lipid media, i.e., on the K_p_ (equation S1) [17,19]. The variation of the parameter (we used fluorescence intensity) upon titration of the peptide with a lipid suspension may result in higher or lower fluorescence intensity, when compared to the one measured in the absence of lipid [19].

(S1)

I_W_ and I_L_ are the fluorescence intensities with all the fluorophore in aqueous solution or in lipid, respectively, γ_L_ is the molar volume of lipid, and [L] is its concentration [17]; the γ_L_ used was 0.763 dm^3^mol^-1^ for vesicles containing POPC [20].

pepR and pepM spectra were collected in a FS920 fluorescence spectrophotometer (Edinburgh Instruments), equipped with a xenon lamp (Xe900). Samples were excited at 492 nm and emission spectra were recorded from 500 to 650 nm (1 and 5 nm excitation and emission bandpasses, respectively).

To quantify the partition extent, DENV C protein-derived peptides (5 µM) were titrated up to 4.5 mM with LUV suspensions of POPC, POPC:POPG 4:1 or POPC:POPG 9:1. Samples were incubated for 10 min after each addition of LUV. The results are presented in Figure S4 and Table S2.


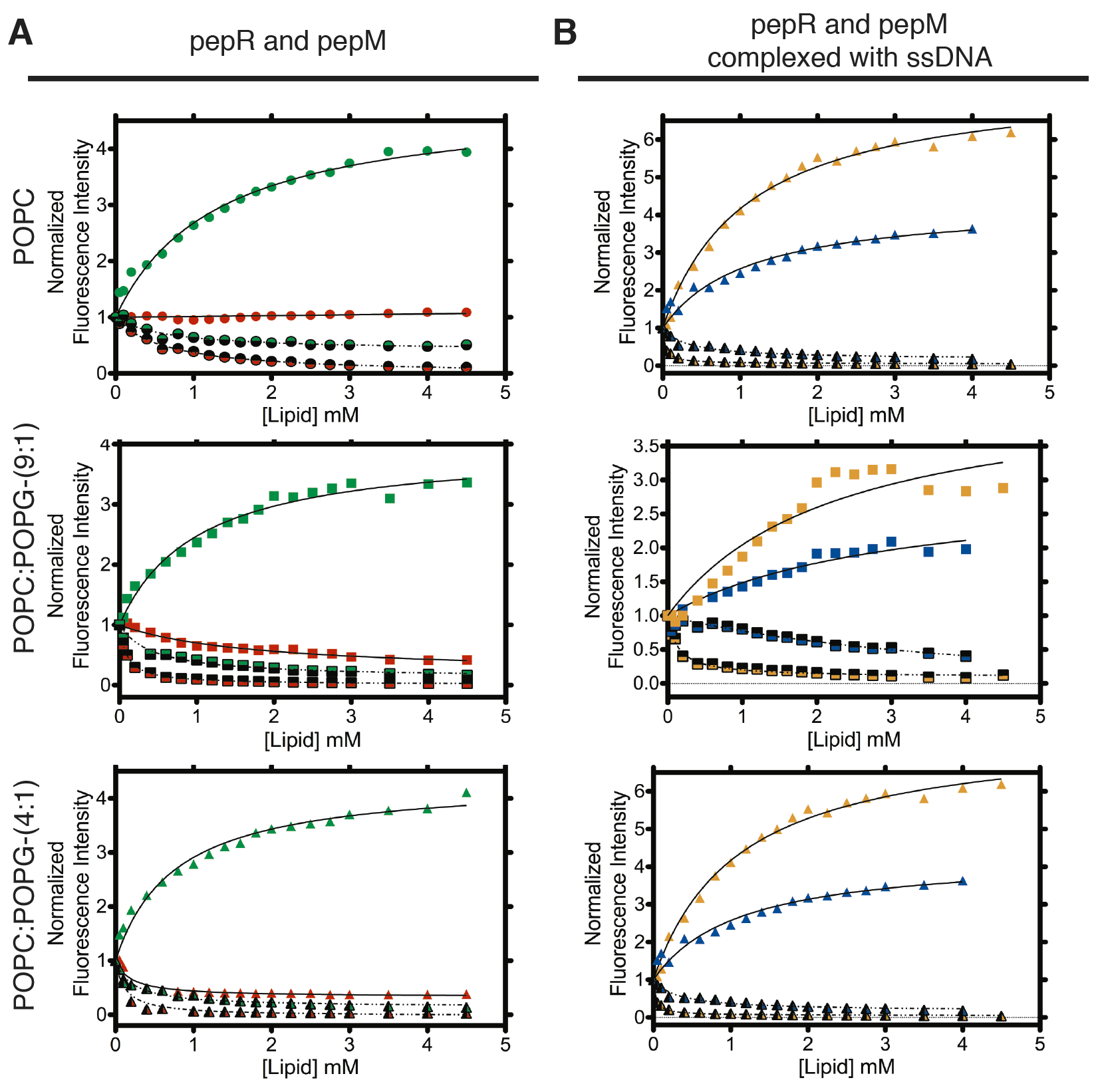


**Figure S4: Membrane partition assays. DENV C protein-derived peptides, pepR and pepM (A), and their ssDNA complexes (B).** A 5 µM fluorescein-labeled pepR (red) or pepM (green) solution was titrated with LUV of POPC (circles) or POPC:POPG (9:1, squares; 4:1, triangles) at pH 7.4 (full colored symbols and full curve) and 5.5 (half colored symbols and dashed curve). Fluorescence emission spectra between 500 and 650 nm with an excitation wavelength of 492 nm were normalized for the value obtained in the absence of LUV. Equation (S1) was used to fit the data to obtain the partition constants, K_p_ (see Table S2). For the ssDNA peptide complexes (pepR complexes – orange; pepM complexes – blue), 1 µM of unlabeled ssDNA was added to a 5 µM peptide solution and then titrated with LUV.

Experiments were carried out both at pH 5.5 and 7.4, with increasing contents of the anionic phospholipid POPG, in order to mimic the endocytic maturation while endocytosis occurs. A newly developed mathematical model was applied to interpret the data, as described elsewhere [16], accounting for simultaneous partition of both the free and complexed peptides, which are in equilibrium with the free ssDNA molecules. Both pepR and pepM show increased lipid membrane affinity upon increasing the fraction of anionic lipid (Table S2). Free pepR does not partition significantly to LUV of the zwitterionic phospholipid POPC, at variance with the pepR-ssDNA complex (Figure S4). The opposite was found for pepM: the free peptide has a more extensive partitioning to the membrane than the complex (Figure S4). The environment pH also influences the lipid partitioning of the peptides. Acidification increases the membrane partitioning of all peptides and their complexes (Table S2), favoring lipid membrane adsorption and consequent lipid membrane translocation and endosomal escape.

**Table S2:** **DENV C protein-derived peptides interaction with lipid vesicles.** Partition constants of the unlabeled (Trp fluorescence), fluorescein-labeled peptides, K_p_ ± SD, and their complexes with ssDNA, K_p,C_ ± SD. Values were calculated using equation (S1).

|  | | | | **(Partition constant ± SD) × 10^3^** | | | |
| --- | --- | --- | --- | --- | --- | --- | --- |
|  | **Lipid** | **pH** | **pepR** | | **pepR-ssDNA** | **pepM** | **pepM-ssDNA** |
| **Unlabeled peptides** | **POPC** | **7.4** | - | | 0.66 ± 0.09 | 4.08 ± 1.02 | 0.87 ± 0.11 |
|  | **POPC:POPG 9:1** | **7.4** | 0.59 ± 0.09 | | 0.47 ± 0.06 | 7.58 ± 2.49 | 1.54 ± 0.26 |
| **Fluorescein labeled peptides** | **POPC** | **7.4** | - | | 0.46 ± 0.09 | 1.00 ± 0.11 | 0.87 ± 0.12 |
|  |  | **5.5** | 2.07 ± 0.16 | | 3.71 ± 0.32 | 2.00 ± 0.40 | 1.96 ± 0.31 |
|  | **POPC:POPG 9:1** | **7.4** | 0.75 ± 0.13 | | 0.58 ± 0.20 | 1.30 ± 0.15 | 0.44 ± 0.12 |
|  |  | **5.5** | 14.02 ± 0.36 | | 9.99 ± 0.85 | 3.00 ± 0.45 | 0.28 ± 0.14 |
|  | **POPC:POPG 4:1** | **7.4** | 6.58 ± 1.40 | | 1.20 ± 0.09 | 1.73 ± 0.19 | 1.12 ± 0.20 |
|  |  | **5.5** | 10.57 ± 1.64 | | 38.5 ± 2.4 | 6.04 ± 0.87 | 4.11 ± 0.67 |

## Membrane translocation assay

The changes in fluorescence emission intensity upon lipid interaction can also be used to evaluate the membrane translocation capability of a molecule or supramolecular complexes. This methodology was previously developed and used by Ferre et al. [21] to evaluate the translocation properties of the antimicrobial peptide BP100. Briefly, kinetic studies were carried out both with LUV and MLV at the same total lipid concentration. In the occurrence of translocation, the kinetics of interaction with the MLV would be slowed down with respect to LUV interaction due to the multiple membrane crossing steps, however reaching the same quantum yield as with the LUV, indicating that the peptides are interacting with the same amount of lipid on the LUV and MLV suspensions. In the absence of translocation, although the lipid concentration is the same in MLV and LUV suspensions, the exposed lipid accessible for interaction (the outer leaflet) is lower in the MLV and the molecule would sense an apparently lower concentration of lipid in this suspension. Therefore, the fluorescence would never increase as much with MLV as with LUV due to less interactions of the molecule understudy with lipids. With LUV, with or without the occurrence of translocation, the entire lipid is accessible to the added molecule at time zero, resulting in a fast interaction kinetics. With molecular translocation on MLV, at time zero only a fraction of the lipid is accessible to the added molecule, resulting in a fast, but partial, increase in fluorescence. As the molecule translocates, more lipid becomes accessible, and a full fluorescence increase is eventually reached at a lower rate. Without translocation on MLV, there is a fast interaction with the accessible fraction of lipid, but no subsequent increase is expected, as no more lipids become accessible. Figure S5 illustrates the methodology using a simplified cartoon.

The kinetic profile of the interaction of both peptides with multilamellar vesicles (MLV) and LUV was registered at controlled conditions where lipid and peptide aggregation does not occur (Figure 5A and 5B in the main text). No membrane translocation occurs with POPC bilayers. However, for pepM this is reverted when anionic lipid is present. pepR is not able to translocate across lipid model membranes by itself or with the ssDNA cargo, even in the presence of POPG. The results show that pepM and pepM-ssDNA complexes are able to translocate across the lipid bilayer, acting as a CPP [14].


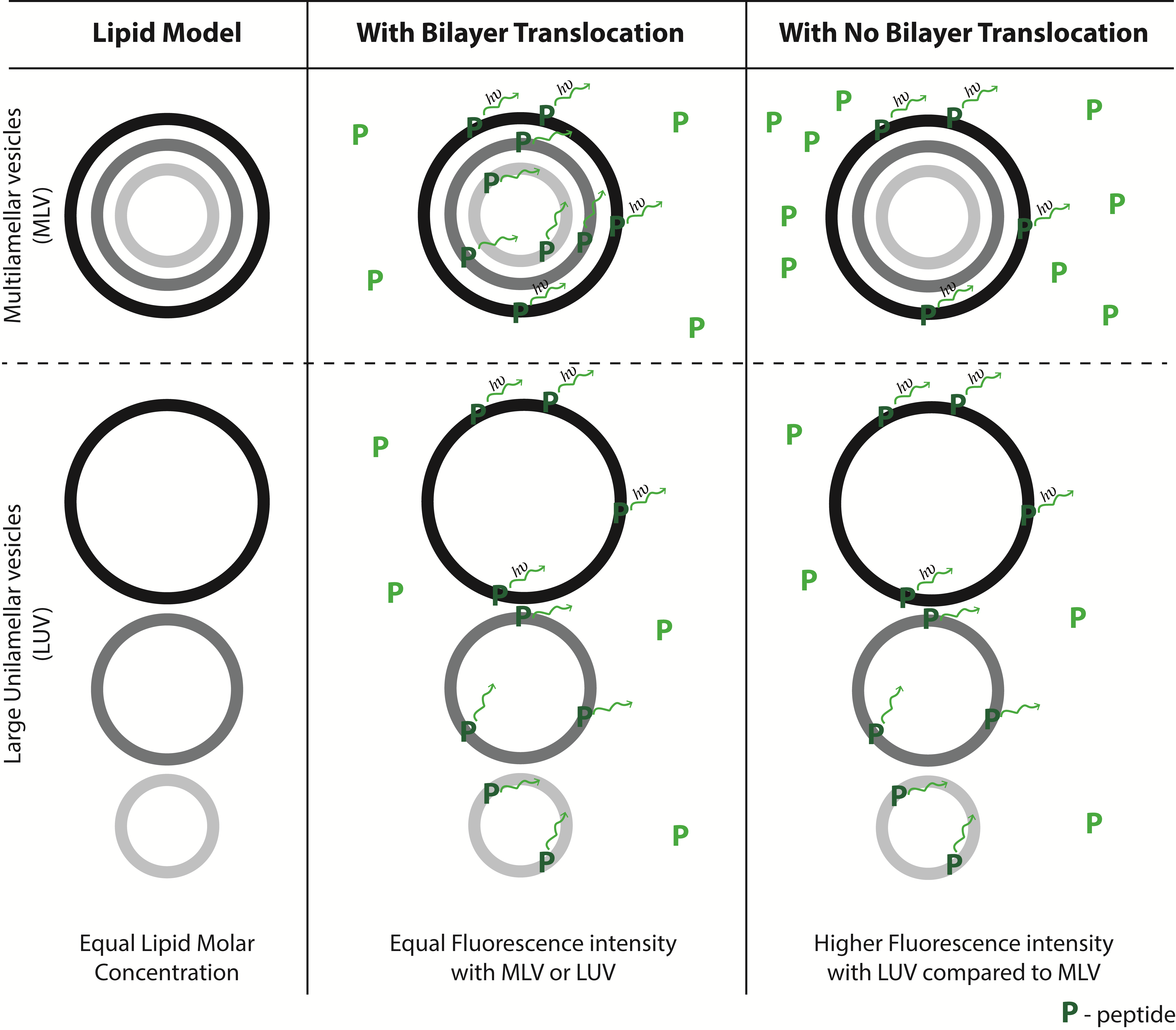


**Figure S5:** Simplified illustration of the methodology followed to conclude on the translocation of peptides across artificial lipid bilayers from Trp residues fluorescence emission. Using MLV and LUV suspensions of equal lipid concentration, equal fluorescence intensity is indicative of membrane translocation.

# References

1. Markoff L, Falgout B, Chang A (1997) A conserved internal hydrophobic domain mediates the stable membrane integration of the dengue virus capsid protein. Virology 233: 105–117. doi:10.1006/viro.1997.8608.

2. Ma L, Jones CT, Groesch TD, Kuhn RJ, Post CB (2004) Solution structure of dengue virus capsid protein reveals another fold. Proc Natl Acad Sci USA 101: 3414–3419. doi:10.1073/pnas.0305892101.

3. Wilkins MR, Gasteiger E, Bairoch A, Sanchez JC, Williams KL, et al. (1999) Protein identification and analysis tools in the ExPASy server. Methods in molecular biology (Clifton, NJ) 112: 531–552. Available: http://eutils.ncbi.nlm.nih.gov/entrez/eutils/elink.fcgi?dbfrom=pubmed&id=10027275&retmode=ref&cmd=prlinks.

4. Sangiambut S, Keelapang P, Aaskov J, Puttikhunt C, Kasinrerk W, et al. (2008) Multiple regions in dengue virus capsid protein contribute to nuclear localization during virus infection. J Gen Virol 89: 1254–1264. doi:10.1099/vir.0.83264-0.

5. Chan DC, Fass D, Berger JM, Kim PS (1997) Core structure of gp41 from the HIV envelope glycoprotein. Cell 89: 263–274.

6. Roy A, Kucukural A, Zhang Y (2010) I-TASSER: a unified platform for automated protein structure and function prediction. Nat Protoc 5: 725–738. doi:10.1038/nprot.2010.5.

7. DeLano W (2008) The PyMOL molecular graphics system (DeLano Scientific LLC, Palo Alto, CA). Wiley-Interscience. 1 pp. Available: http://scholar.google.com/scholar?q=related:DLqWH9so7N4J:scholar.google.com/&hl=en&num=30&as_sdt=0,5.

8. Fields GB, Noble RL (1990) Solid phase peptide synthesis utilizing 9-fluorenylmethoxycarbonyl amino acids. Int J Pept Protein Res 35: 161–214.

9. Fritzsche M, Mandenius C-F (2010) Fluorescent cell-based sensing approaches for toxicity testing. Anal Bioanal Chem 398: 181–191. doi:10.1007/s00216-010-3651-6.

10. Greim H, Snyder R (2008) Toxicology and risk assessment. Wiley-Interscience. 1 pp. Available: http://books.google.com/books?id=gZbnUpN48xgC.

11. Alves CS, Melo MN, Franquelim HG, Ferre R, Planas M, et al. (2010) Escherichia coli Cell Surface Perturbation and Disruption Induced by Antimicrobial Peptides BP100 and pepR. J Biol Chem 285: 27536–27544. doi:10.1074/jbc.M110.130955.

12. Netsawang J, Noisakran S, Puttikhunt C, Kasinrerk W, Wongwiwat W, et al. (2010) Nuclear localization of dengue virus capsid protein is required for DAXX interaction and apoptosis. Virus Res 147: 275–283. doi:10.1016/j.virusres.2009.11.012.

13. Murphy BR, Whitehead SS (2011) Immune Response to Dengue Virus and Prospects for a Vaccine. Annu Rev Immunol 29: 587–619. doi:10.1146/annurev-immunol-031210-101315.

14. Henriques ST, Melo MN, Castanho MARB (2006) Cell-penetrating peptides and antimicrobial peptides: how different are they? Biochem J 399: 1. doi:10.1042/BJ20061100.

15. Jarver P, Mäger I, Langel Ü (2010) In vivo biodistribution and efficacy of peptide mediated delivery. Trends Pharmacol Sci 31: 528–535. doi:10.1016/j.tips.2010.07.006.

16. Freire JM, Veiga AS, la Torre de BG, Andreu D, Castanho MARB (2013) Quantifying molecular partition of cell-penetrating peptide-cargo supramolecular complexes into lipid membranes: optimizing peptide-based drug delivery systems. J Pept Sci 19: 182–189. doi:10.1002/psc.2477.

17. Santos NC, Prieto M, Castanho MARB (2003) Quantifying molecular partition into model systems of biomembranes: an emphasis on optical spectroscopic methods. Biochim Biophys Acta 1612: 123–135. doi:10.1016/S0005-2736(03)00112-3.

18. Matos PM, Franquelim HG, Castanho MARB, Santos NC (2010) Quantitative assessment of peptide–lipid interactions. Biochim Biophys Acta 1798: 1999–2012. doi:10.1016/j.bbamem.2010.07.012.

19. Castanho MARB, Fernandes MX (2005) Lipid membrane-induced optimization for ligand–receptor docking: recent tools and insights for the “membrane catalysis” model. Eur Biophys J 35: 92–103. doi:10.1007/s00249-005-0007-9.

20. Nagle JF, Wiener MC (1988) Structure of fully hydrated bilayer dispersions. Biochim Biophys Acta 942: 1–10.

21. Ferre R, Melo MN, Correia AD, Feliu L, Bardaji ER, et al. (2009) Synergistic Effects of the Membrane Actions of Cecropin-Melittin Antimicrobial Hybrid Peptide BP100. Biophys J 96: 1815–1827. doi:10.1016/j.bpj.2008.11.053.
